# Enzyme-mediated formulation of stable elliptical silver nanoparticles tested against clinical pathogens and MDR bacteria and development of antimicrobial surgical thread

**DOI:** 10.1186/s12941-017-0216-y

**Published:** 2017-05-16

**Authors:** Rupak Thapa, Chintan Bhagat, Pragya Shrestha, Suvash Awal, Pravin Dudhagara

**Affiliations:** 1 Department of Biotechnology, Veer Narmad South Gujarat University, Surat, Gujarat 395007 India; 2 Department of Biosciences (UGC-SAP-DRS-II), Veer Narmad South Gujarat University, Surat, Gujarat 395007 India

**Keywords:** Silver nanoparticles (AgNPs), Antimicrobial activity, Multi-drug resistant (MDR), Surgical thread

## Abstract

**Background:**

Silver nanoparticles (AgNPs) are believed to be emerging tool against various infectious diseases including multi-drug resistant (MDR) bacteria. In the present study, in vitro synthesis of AgNPs was optimized using 1:50 ratio of macerozyme (25 μg/μl) and 1 mM AgNO_3_ incubated at 80 °C for 8 h. AgNPs were characterized by UV–Visible spectroscopy, dynamic light scattering (DLS), scanning electron microscopy, energy-dispersive X-ray spectroscopy, transmission electron microscopy (TEM) and X-ray diffraction (XRD).

**Results:**

Characterization studies suggest the synthesis of elliptical, stable and crystalline AgNPs with an average size of 38.26 ± 0.4 nm calculated using TEM. The XRD pattern revealed the face-centered-cubic (fcc) form of metallic silver. Good shape integrity and dispersion of AgNPs after 1 year of incubation confirmed their stability. AgNPs were exibited the antimicrobial property against ten pathogenic bacteria, three molds and one yeast. The AgNPs also revealed remarkable antimicrobial activity against three MDR strains i.e. Extended spectrum beta-lactamase positive *Escherichia coli*, *Staphylococcus aureus* (MRSA) and Teicoplanin resistant *Streptococcus Pneumoniae.* The AgNPs coated surgical threads (suture) were revealed the remarkble antibacterial activity against three MDR strains. This is the first report to synthesize antimicrobial elliptical AgNPs using enzymes.

**Conclusion:**

The results suggest the possibilities to develop the nanoparticles coated antimicrobial medical fabric to combat against MDR infection.

**Electronic supplementary material:**

The online version of this article (doi:10.1186/s12941-017-0216-y) contains supplementary material, which is available to authorized users.

## Background

The silver and gold nanoparticles have been largely explored in biomedical sector due to its great potential as a broad spectrum antimicrobial agent [1–4]. Despite of its wide use, antibacterial activity of silver nanoparticles is poorly understood with few implicit mechanisms including (1) generations of reactive oxygen species (ROS) intracellularly and/or extracellularly, (2) cellular uptake of silver ions (3) cascade of intracellular reactions and (4) interactions between nanoscale silver and cell membranes [5, 6]. Recently, Hsueh et al. [7] reported the release of Ag+ ions intracellular or extracellular from the AgNPs, highlights the precise antimicrobial mechanism. Based on these mechanisms, AgNPs have been employed and established as an antibacterial agent in wound treatment, medical devices, cosmetics, water purification, air treatment and clothing [8, 9]. Presently, >30% of AgNPs are utilized by the healthcare sector due to impressive demand of antimicrobial consumable stuff. Thus, the growing demand of antimicrobial materials in healthcare applications is projected to drive silver nanoparticles market to reach USD 2.45 billion by 2022 (http://www.grandviewresearch.com/press-release/global-silver-nanoparticles-market). This ongoing demand of the AgNPs will promote the exploration of various new synthesis routes.

Nanobiotechnological production of nanoparticle using biomolecules offers the characteristic of specificity, benign nature, viability and conceivability for functionalization [2]. Biocatalysis dependent nanoparticles synthesis was found to be more pharmacologically active than chemical route formulation and holds promise for amended biocompatible nanoparticles production [10]. Few green approaches have been tested for metal nanoparticles synthesis, which includes the use of plant extract, enzyme, biomolecule, biosurfactant and microorganisms [11–15]. In vitro synthesis process is new developed concept which eliminates the culturing of the microorganisms and extraction of their metabolites/macromolecules. Several enzymes have been explored for lab scale as well as bulk production of AgNPs including keratinase, nitrate reductase, alpha-amylase and sulfite reductase [16–19]. Enzyme-mediated nanoparticle formation is widely utilized methods as it is simple, eco-friendly, inexpensive and easily scaled-up process for bulk production of functionalized and stable nanoparticles [16–18]. The size and shape controlled synthesis of the AgNPs is the key issues to determined their efficacy and applications. Such controlled synthesis of AgNPs can be formulated through the optimization of process variables including pH, temperature, reaction time, enzyme concentration and substrate concentration.

Biosynthesis of the size and shape controlled nanoparticles are yet the challenging task for the nanotechnologiest. The size of the AgNPs determines their effectiveness against pathogenic bacteria. Smaller than 50 nm of AgNPs are reported as a best antimicrobial weapon against bacterial pathogens [20]. The shape of AgNPs also play the significant role to kill the microorganisms, sphere-shaped nanoparticles are considered to be the best-suited for antimicrobial applications in either colloidal form or immobilized state [21]. Elliptical or oval AgNPs may also more antimicrobial than other reported shape due to more surface area to interact with cell membrane and ease to inter into cell. Morphological variation of the AgNPs is not only the determinant of microbial susceptibility, but the microbial cell structure and type (i.e. Gram positive and negative), are also play a significant role [22]. Earlier studies of AgNPs as bactericides have been largely explored through the model microbial species—*Escherichia coli* [23]. The outputs of such experiments are not adequate to confirm the AgNPs as antimicrobials. Additionally, the susceptibility of MDR bacteria against antimicrobials is very negligible than the non-MDR bacteria. So the multiple non-MDR and MDR bacterial species need to test against the AgNPs to establish it as antimicrobials.

Currently, the outbreak of various MDR strains globally at the alarming rate resulted in treatment difficulties which have imposed a burden on health care systems and simultaneously intensifying the need for new antimicrobial agents [24]. Recently, WHO list out the 12 MDR bacterial species (http://www.who.int/mediacentre/news/releases/2017/bacteria-antibiotics-needed/en/) that pose a greatest threat to human health suggest the prompt need of new therapy to manage this antimicrobial crisis. The discovery of the new antimicrobials is quite slower than the emerging rate of the antimicrobial resistance among the bacteria. Provisionally, the use of the metal nanoparticles to achieve the success in antimicrobial crisis can be a viable option. Due to the interaction of nano-silver with multiple microbial target sites, it is expected to be feasible and promising alternative against most common MDR strains such *E. coli*, *Staphylococcus aureus* and *Streptococcus pneumoniae*. Moreover, the synergistic activity of AgNPs coupled with antibiotics is also an advisable approach to combat against various MDR strains. Therefore, stable AgNPs are effective bactericidal materials due to its combined effects with antibacterial agents and are the robust ammunition against the MDR strains [25].

In present research, attempt was made to in vitro synthesis of AgNPs using macerating enzymes. AgNPs characterization was investigated to confirm the size, shape, and nature. The antimicrobial activity of AgNPs was studied against pathogenic bacteria, yeast and molds. The key investigation was carried out by testing the antimicrobial activity against three MDR bacterial species *E. coli*, *S. aureus* and *S. pneumoniae*.

## Methods

### Materials

Macerating enzymes, Macerozyme R-10, Nutrient agar, Blood agar, Chocolate agar and MacConkey agar, Mueller–Hinton (MH) agar and potato dextrose agar (PDA) were purchased from Hi-Media Laboratories Pvt. Ltd. Mumbai, India. Silver nitrate (AgNO_3_) was acquired from S.D. Fine-Chem. Ltd. Mumbai, India. Throughout the experiment, MiliQ (Millipore^®^) water was used for solutions preparation. The antibiotics sensitivity tests were verified using antibiotics disc purchased from Hi-Media Laboratories Pvt. Ltd. Mumbai, India. The identification of clinical cultures was carried out by VITEK^®^2 system (Biomerieux Inc. Hazelwood, Mo.) at Anand Laboratory, Surat, India.

### Synthesis of AgNPs

Total 20 reaction systems were prepared using different concentration of macerozymes and AgNO_3_. Different volumes of macerozyme (25 μg/μl solution) i.e. 20, 100, 200, 500, and 1000 µl were added separately in 5 ml AgNO_3_ solution of varying concentration of 0.1, 0.5, 1 and 2 mM and were incubated at 37, 60, 80, and 90 °C for 2–8 h. The pH of all the reaction systems was adjusted 7.2 ± 0.2. After every 2-h interval, a reduction of silver ions was confirmed by the color changes and verifies the time required for the formation of AgNPs.

### Characterization of nanoparticles

#### UV–Vis spectroscopy

Formation of AgNPs indicated by the color changes of the reaction mixture from colorless to pinkish-red due to the bioreduction of the silver ions using enzymes. The synthesis of AgNPs was assured by scanning the absorption spectra of the reaction mixture over the range of 300–800 nm wavelengths with UV–Vis spectrophotometer (UV-1800, Shimadzu, Japan) using dual beam operated at 1 nm resolution. The optimum result indicated reaction was subjected to further analysis.

#### Dynamic light scattering

Zetasizer (Nano-ZS, Malvern Instruments, USA) was used to monitor the size distribution profile of synthesized colloidal AgNPs using MiliQ water as a dispersant (refractive index = 1.330, viscosity = 0.8872 cP). The size calculation was carried out using colloidal sample and means value of three readings was considered.

#### Scanning electron microscopy and elemental analysis

Colloidal AgNPs dried at 60 °C, and analyzed by SEM (JEOL JSM-6360LV), operating at an accelerating voltage of 15.0 kV under high vacuum. The dried AgNPs sample was applied to the carbon-coated copper grid for measurement of elemental composition profile of the sample using EDX equipped with SEM.

#### TEM analysis

Colloidal AgNPs were further analyzed by the TECNAI G^2^ TEM instrument (FEI Corp. Hillsboro, USA) operating at 80 kV, and the image was taken using TIA software after drop coating with 50 μl of nanoparticles on a carbon-coated copper grid of 300 mesh size. After 1 year storage at 37 °C, colloidal AgNPs were again visualized using TEM to check the agglomeration.

#### X-ray diffraction analysis

Dried powder of synthesized AgNPs analyzed by diffractometer on X’Pert Pro PANaltyical, USA operated at 45 kV and 40 mA current at 25 °C temperature and diffraction pattern was recorded by CuKα1 radiation with λ of 1.54 Å, step size 0.017 in the region of 2θ from 20 to 100.

### Evaluation of antimicrobial activity

Out of six Gram-positive bacteria, four i.e. *Micrococcus luteus* NCIM 5262, *Bacillus subtilis* NCIM 2920, *Bacillus cereus* NCIM 5557, and *Bacillus megaterium* NCIM 5334 as well as four molds include *Aspergillus niger* NCIM 1358, *Mucor racemosus* NCIM 1334, *Penicillium chrysogenum* NCIM 1333, *Rhizopus stolonifer* NCIM 1139 were purchase from National Collection of Industrial Microorganisms (NCIM), Pune, India. These laboratory control strains of bacteria and molds were maintained on Nutrient agar and PDA respectively. *S. aureus* and *S. pneumoniae* were isolated on Blood agar from the blood and throat swab respectively. *E. coli, Klebsiella pneumoniae*, *Proteus vulgaris*, *Pseudomonas aeruginosa*, were isolated using Blood agar, Chocolate agar and MacConkey agar from urine, throat swab, urine and sputum respectively. The *Candia albicans* was isolated form viginal swab on PDA.

Antimicrobial activity of AgNPs was analyzed using disc diffusion method against clinical pathogenic bacteria, yeast and molds. The pure cultures of all bacteria and yeast were grown on MH liquid medium. The active culture of bacteria and yeast were uniformly swabbed onto the MH agar plates using sterile cotton swabs, pre-soaked with 20 μl of each culture (A_620 nm_ = 0.85). After incubation at 37 °C for 24 h, the zone of inhibition (ZOI) was measured in millimeter and result was recorded in triplicate experiments. Whereas, each sporulated mold were grown on PD agar plates using spores sprinkling method by placing the plate containing one week old growth of mold and tapped ten times on fresh PD agar plate aseptically and incubate 5 days at 30 °C to obtain an adequate growth. Subsequently, paper discs were impregnated with 20 μl of AgNPs, AgNO_3_, and sterile H_2_O and laid aseptically over the pre-inoculated agar plates.

### Activity against MDR bacteria

The ESBL positive *E. coli*, *S. aureus* (MRSA), Teicoplanin resistance *S. pneumoniae* were isolated from urine, blood and throat swab of pediatric sample respectively. Blood agar Chocolate agar and MacConkey agar were used for the isolation. Identification were carried out by using VITEK^®^2 system. For the verification of the MDR, the antibiotic susceptibility of each strain was performed using disc diffusion test on MH agar against various antibiotics suggested by guideline (2016) of National Committee for Clinical Laboratory Standards (NCCLS), now Clinical and Laboratory Standards Institute (CLSI). Susceptibility of 20 μl of AgNPs, AgNO_3_, and sterile H_2_O was also tested as discussed in earlier section.

### Development of nano-coated surgical threads

The nanocoated surgical threads were developed by dipping 30 mm long threads into colloidal AgNPs followed by drying at 45 °C and wash aseptically with sterile distilled water. Nanocoated threads were placed in MH agar plates, pre-streaked with three MDR strains and antimicrobial activities were tested as explained earlier section.

## Result

### Synthesis of AgNPs formation

Out of 20 different reactions, four reactions containing 100 µl of macerozyme solution (25 μg/μl) and 5 ml of 1 mM AgNO_3_ incubated at four different temperature i.e. 37, 60, 80 and 90 °C were selected and among them the best AgNPs synthesis was observed at 80 °C temperature after 8 h. As the time and temperature increased in all four reaction mixtures, the reduction of the silver ions was increased and corresponding orange color was also intensify. However, UV–Vis spectroscopic analysis indicated the narrow bell shaped graphs with highest absorbance at 425 nm after 8 h treatment at 80 °C was selected as a best reaction **(**Additional file 1 **).**

### Characterization of AgNPs

**Fig. 1:**
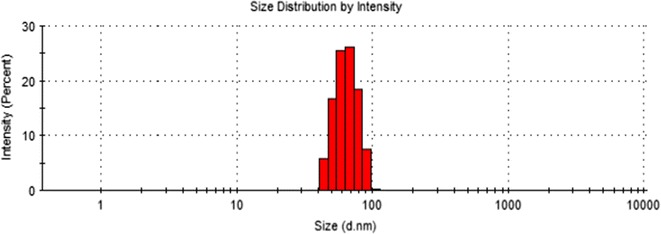
Size distribution measurement of colloidal AgNPs by DLS

**Fig. 2:**
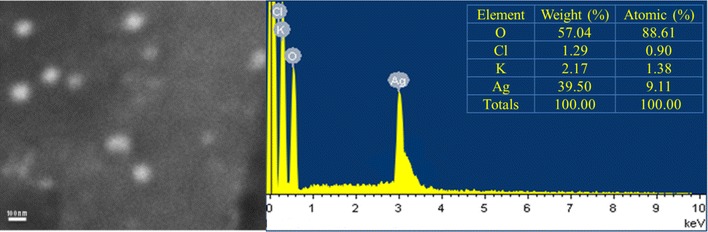
Scanning electron micrographs (SEM) of AgNPs and elemental profiling using EDX. Satellite *table* indicates the mass composition of elements

**Fig. 3:**
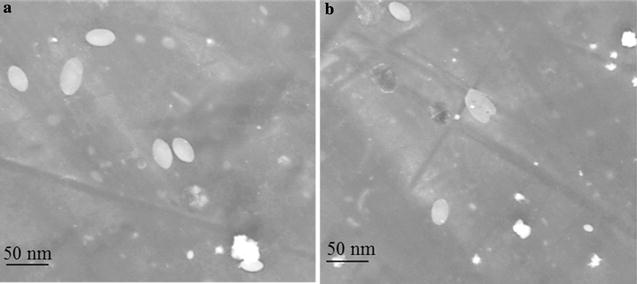
Confirmation of elliptical shape of AgNPs by TEM **a** in fresh and **b** 1-year-old colloidal sample

**Fig. 4:**
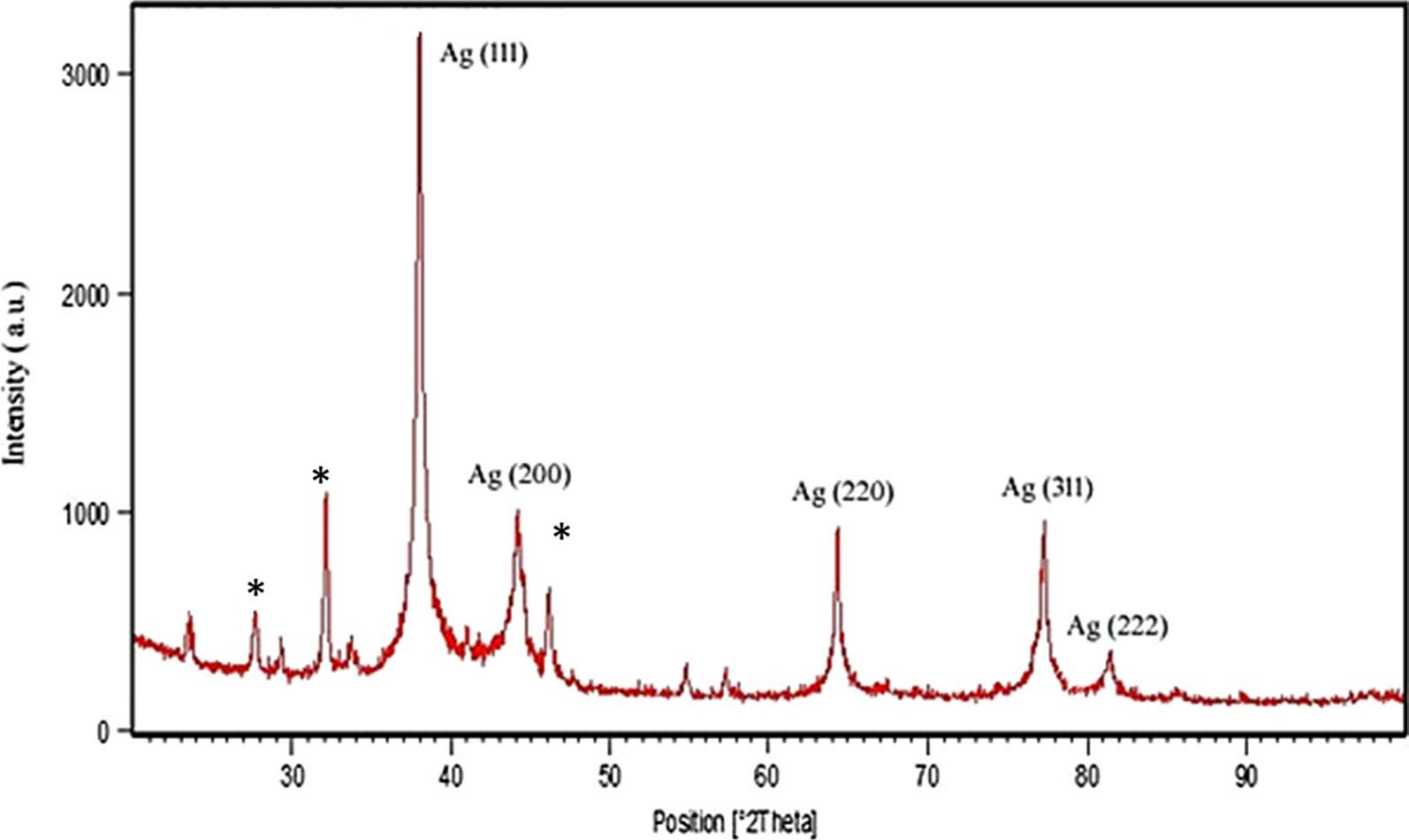
XRD pattern of AgNPs

Optimized reaction based on UV-Vis spectroscopic analysis, AgNPs were subjected for particle size analysis. The average size of ~90% AgNPs was 63.65 ± 12.71 nm by DLS with a good dispersity **(**Fig. 1 **).** SEM results suggest the elongated morphology of the AgNPs. Energy dispersive X-ray spectrum of AgNPs revealed the clear and heightened peak at 3 keV correspond to the binding energies of Ag Lα, which is the characteristics peak of nano-silver **(**Fig. 2) [26]. Elemental composition profile indicates the relatively high purity of the particles with high concentration of silver i.e. 39.50% by weight. The result of the TEM confirmed elliptical shape of individual particles with average diameter of 38.26 ± 0.4 nm in fresh and 1-year-old sample **(**Fig. 3 **)**. A very slight clump was observed in old sample; however, the visible clumps were absent. To the best of our knowledge, this is the first report to formulat the elliptical shaped particles using biological route. The XRD analysis confirmed the presence of peaks at 2θ = 38°, 44°, 64°, 77° and 81°, which coincide to **(**111**)**, **(**200**)**, **(**220**)**, **(**311**)** and **(**222**)** indicating planes of silver respectively **(**JCPDS, standard file no. 04-0783**) (**Fig. 4 **)**. To calculate the crystalline (grain) size form the XRD data, we use standard Debye–Scherrer method for better conformation of crystalline size. The average crystalline size of our prepared sample was 36.01 ± 1.4 nm (Table 1). The size of AgNPs measured by TEM and XRD are supportive but differs from the DLS analysis due to the brownian motion and elliptical shape of particles that lead to the more diverse results of DLS than TEM and XRD.

**Table 1:** Crystalline size of AgNPs calculated using Debye–Scherrer’s equation

2θ (deg)	θ	Planes-hkl	FWHM (deg)	Crystalline size (nm)	Average crystalline size (nm)
38	19	111	0.041	35.70	36.01 ± 1.4
44	22	200	0.043	34.76	
64	32	220	0.047	34.77	
77	38.5	311	0.048	36.89	
81	40.5	222	0.048	37.97	

### Antimicrobial activity

**Fig. 5:**
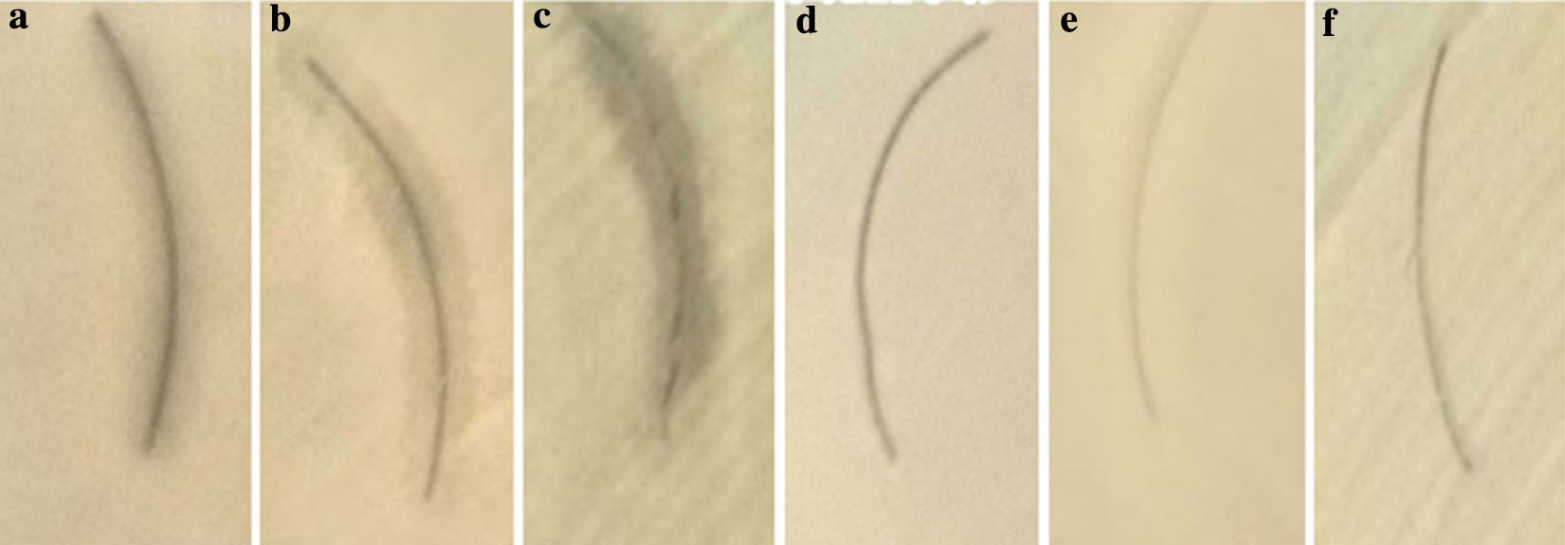
Antimicrobial activity of AgNPs coated surgical suture against **a** ESBL positive *E*. *coli*, **b** *S. aureus* MRSA, **c** Teicoplanin resistant *S. pneumoniae*. Control: Sterile H_2_O treated suture tested against **d** ESBL positive *E*. *coli* (E) *S. aureus* MRSA **f** Teicoplanin resistant *S. pneumoniae*

The efficiency of antibacterial activity of AgNPs was assessed as size of ZOI against the ZOI of AgNO_3_ solution and sterile H_2_O. AgNPs were found to have better antimicrobial activity against *B. subtilis*, *M. leutus*, *P. aeruginosa*, *B. megaterium* and *S. aureus* having a zone of inhibition of more than 15.0 ± 0.0 mm. The AgNPs showed remarkable antifungal activity against all the tested strains among which, maximum activity was seen against *R. stolonifer* with 14.0 ± 1.0 mm ZOI and minimum activity was seen against *C. albicans* with 12.0 ± 0.0 mm ZOI. The percentage increases in inhibition zone area for the AgNPs against *P. chrysogenum* NCIM 1333 was highest i.e. 96% followed by *A. niger* NCIM 1358 (43.12%), *E. coli* (32.45%) and *P. aeruginosa* (31.82%). For other tested microorganisms, the percent of increases of inhibition zone area were ranging from 15.62 to 31.65% **(**Table 2 **)**. AgNPs ZOI against ESBL positive *E. coli* was 10.5 ± 0.50 mm, which is far greater than the most of the tested antibiotics. *S. aureus* (MRSA) showed the 13.5 ± 0.5 mm zone which is 2.5 ± 0.5 mm greater than the AgNO_3_. Teicoplanin resistant *S. pneumoniae* was exibited the 13.16 ± 0.28 mm ZOI (Table 3). From the study, the comparative effectiveness of AgNPs and AgNO_3_ against MDR strains was clearly observed. AgNPs showed clear zone of inhibition that was greater than various tested first and second generation antibiotics. The remarkable antimicrobial activity of AgNPs treated surgical threads against ESBL positive *E*. *coli*, *S. aureus* (MRSA) and Teicoplanin resistant *S. pneumoniae* indicates the firmly coating of AgNPs on the threads (Fig. 5).

**Table 2:** Antimicrobial activity of colloidal AgNPs against various species of pathogenic bacteria, yeast and molds

Sr. no.	Microorganisms	ZOI of 20 μl of AgNPs (mm) (y)	ZOI of 20 μl of 1 mM AgNO_3_ (mm) (x)	ZOI of 20 μl of H_2_O (mm)	Increase in the ZOI area (%)^a^
1	*E. coli*	12.66 ± 0.28	11.00 ± 0.0	0 ± 0	32.45
2	*S. pneumoniae*	14.33 ± 0.57	13.16 ± 0.28	0 ± 0	18.57
3	*K. pneumonia*	14.16 ± 0.28	13.0 ± 0.5	0 ± 0	18.64
4	*P. vulgaris*	14.00 ± 0.5	12.33 ± 0.28	0 ± 0	28.92
5	*S. aureus*	15.0 ± 0.0	13.16 ± 0.28	0 ± 0	29.91
6	*P. aeruginosa*	15.5 ± 0.5	13.5 ± 0.5	0 ± 0	31.82
7	*B. cereus* NCIM 5557	12.50 ± 0.5	11.16 ± 0.28	0 ± 0	25.45
8	*B. megaterium* NCIM 5334	15.33 ± 0.28	13.5 ± 0.5	0 ± 0	28.94
9	*M. luteus* NCIM 5262	15.5 ± 1.0	14.0 ± 0.5	0 ± 0	22.57
10	*B. subtilis* NCIM 2920	16.0 ± 1.0	14.0 ± 0.5	0 ± 0	30.61
11	*C. albicans*	12.0 ± 0.0	11.16 ± 0.28	0 ± 0	15.62
12	*A. niger* NCIM 1358	13.16 ± 0.28	11.0 ± 0.5	0 ± 0	43.12
13	*M. racemosus* NCIM 1334	13.0 ± 0.5	11.33 ± 0.28	0 ± 0	31.65
14	*P. chrysogenum* NCIM 1333	14.0 ± 1.0	10.0 ± 0.5	0 ± 0	96.00
15	*R. stolonifer* NCIM 1139	14.0 ± 1.0	12.5 ± 1.0	0 ± 0	25.44

All experiments were performed in triplicate

^a^The mean surface area of the growth inhibition zone (mm^2^) was calculated for AgNPs and AgNO_3_ from the mean diameter. The percent of increases of inhibition zone area for the AgNPs against different microorganism were calculated as (y^2^−x^2^)/x^2^ × 100, where x and y are the inhibition zone for 20 μl of AgNO_3_ and 20 μl of AgNPs respectively

**Table 3:** Antibiotic-resistance profile of MDR strains and AgNPs susceptibility test

Bacteria	Tested antibiotics	Disk content (µg)	ZOI (mm)	Antibiotics susceptibility interpretation^a^
ESBL+ve *E. coli*	Nalidixic acid	30	6.0 ± 0.50	R
	Doxycycline	30	16.16 ± 0.28	S
	Norfloxacin	10	8.0 ± 0.50	R
	Nitrofurantoin	30	15.0 ± 0.50	I
	Cefixime	30	6.0 ± 0.50	R
	Cefixime/clavulanate	5/10	8.0 ± 0.50	R
	Cefoperazone	75	6.0 ± 0.0	R
	Cefoperazone/tazobactam	75/10	19.5 ± 1.0	S
	Ceftazidime	30	7.0 ± 1.0	R
	Cetazidime/clavulanate	30/10	14.0 ± 0.0	I
	Chloramphenicol	30	15.16 ± 0.28	I
	Cefprozil	30	6.5 ± 0.50	R
	Cefepime	30	10.5 ± 0.50	R
	AgNPs	2.16^b^	10.5 ± 0.50	–
	AgNO_3_	3.4	9.16 ± 0.28	–
	Sterile H_2_O	20 µl	0 ± 0	–
*S. aureus* (MRSA)	Oxacillin	1	6.66 ± 0.28	R
	Cefoxitin	30	19.33 ± 0.57	R
	AgNPs	2.16^b^	13.5 ± 0.5	–
	AgNO_3_	3.4	11.0 ± 0.0	–
	Sterile H_2_O	20 µl	0 ± 0	–
*S. pneumoniae* MDR	Teicoplanin	30	10.0 ± 0.0	R
	AgNPs	2.16^b^	13.16 ± 0.28	–
	AgNO_3_	3.4	11.0 ± 0.0	–
	Sterile H_2_O	20 µl	0 ± 0	–

All experiments were performed in triplicate

*R* resistant, *I* intermediate, *S* susceptible

^a^The antibiotics susceptibility interpretation was based on CLSI guideline-2016

^b^20 μl of 1 mM AgNO_3_ (MW 169.87) contains 3.4 μg AgNO_3_ and the percentage of silver is 63.5% in nanoparticles (i. e 2.16 μg)

## Discussion

AgNO_3_ reduction and AgNPs formation was found to temperature and time dependent. The increase in the reaction temperature and the time, increase the accessibility of the silver ions for the rapid reduction. The synthesis of AgNPs at elevated temperature is due to the accessibility of thiol groups of macerating enzymes. High temperature unfolds the structure of enzymes and makes an easy availability of thiol groups for the bioreduction of metal (i.e. silver) [27, 28]. However, there are many possibilities for reduction using enzymes. The optimum temperature was 80 °C and time was 8 h. The further increase up to 90 °C caused the broadening of the peak suggesting the bigger and heterogeneous particles formation that led the decrease in absorption [29]. The sharpness of the absorbance peak is dependent to size of the AgNPs because the particle size may be smaller at the higher temperature consequently narrow and sharp SPR band of AgNPs was observed [30]. Beside temperature, reaction time is another factor to determine the controlled synthesis. The steady increasing of absorbance spectrum with increasing the reaction time resulted in the colorless reaction mixture shifts to pinkish-orange with the duration of incubation indicates the increased concentrations of the AgNPs. Thus, the rate of synthesis can be accelerated by increasing the temperature and contact time [31].

Size distribution profile of the colloidal nanoparticles showed narrow monodispersity with an average size of 63.65 ± 12.71 nm by DLS. Nucleation of the silver ions and growth of nanoparticles is the key processes to determine the size. In biosynthesis route the amino and carboxyl group are usually involved into nucleation process. However, the mechanism of biogenic AgNPs is poorly understood. Earlier reports of enzymes assisted AgNPs synthesis suggested the involvement of various amino acids and their functional groups for the AgNPs formation [32, 33]. The result of EDX proved the presence of nearly 40% silver and 60% other trace metals **(**O, K, Cl**)** by weight; this is primarily due to the biogenic root of synthesis. Biological derived impurities like carbon, oxygen, potassium are usually involves in stabilizing the particles by capping on the surface of AgNPs [34]. Absence of oxides indicated the sample contain pure silver. TEM image confirmed elliptical shape and average size of particles is 38.26 ± 0.4 nm. TEM also indicate the good stability of particles with negligible aggregation after 1 year may be due to the presence of Cl ions. The XRD pattern reveals the AgNPs correspond to face-centered cubic (fcc) crystalline phase. Few minor unassigned peaks (*) were due to the formation of the crystalline bioorganic compounds by the enzyme [35].

Antimicrobials resistance (AMR) in pathogenic microorganisms is ever challenging global issue. A wide range of antimicrobial compounds and their combination are used to control AMR in the MDR bacteria and the searching of new antimicrobials for MDR species are yet ongoing practices. One of the best substitutions to combating the resistant problem in different microbes is use of metal nanoparticles due to the multifaceted antimicrobial mechanisms of metals nanoparticles against wide range of microbes [36, 37]. Silver is known to be used for centuries as a potent antimicrobial weapon because of its growth hindrance abilities against a diverse microorganism by exerting its effect at multiple site. AgNPs synthesized by various routes and of different size and shape was tested against diverse clinical pathogenic MDR strains [4, 8, 12, 13, 16, 25, 31]. Due to failure of first and second line antibiotics, the exploration of AgNPs against various MDR strains is the new emerging trend. These suggest the further exploration of silver or other nanoparticles for treatment of various MDR, extensively drug-resistant (XDR) and pan drug resistance (PDR) pathogens. The remarkable antimicrobial activity of 2.1 µg of AgNPs was reported against *S. aureus*, *B. subtilis*, *P. aeruginosa*, *P. vulgaris*, *M. racemosus*, *P. chrysogenum* and *R. stolonifer.* The minimum inhibitory concentration of AgNPs is found to be varies in literature and it depends on coating medium, route of synthesis, types of strains, different size and shape of nanoparticles [4, 8, 16, 30, 38–40]. Result of antimicrobial activity against *E*. *coli* ESBL positive, *S. aureus* (MRSA) and MDR *S. pneumoniae* were also very excellent. The antimicrobial activities against all the Gram-negative and Gram-positive bacteria are more than 12.50 ± 0.5 which is far better than the earlier report [20]. The toxicity of AgNPs is due to the release of intracellular Ag ions in bacterial cell but its orders of magnitude is different into different bacterial species because the intracellular bioavailability of Ag ions liberated from AgNPs is bacterial strain-specific [41]. Moreover, extracellular secretion i.e. organic acids, peptides, biosurfactants and cellular uptake of Ag ions via cell-nanoparticles interaction my affects the toxicity. Additionally, the AgNPs-cell contact is the key phenomenon to determine the toxicity of nanoparticle [41]. The formation of biocorona by hydrogen bonding and hydrophobic interactions between cellular peptides/proteins and AgNPs may varies on cell to cell, consequently the toxicity will be varies among the different microbial cell [42]. Thus, the binding behaviors and kinetics of AgNPs with proteins are important aspects in realizing the toxicity of AgNPs in biosystem [43]. The size and shape determine the surface-to-volume ratio of AgNPs and impacting on its effectiveness. AgNPs are readily interact with organic materials and mounted in fabric for slow release to exert their long-lasting antimicrobial effect. Thus, the safe use and enduring antimicrobial properties can be achieved by tethering the AgNPs on organic nanosheets [44]. Antimicrobial property of nano-coated surgical threads is a key outcome of the present study which is promising tool to control the spreading of MDR infections over a long time. The nano-silver coated nylon thread is useful in the healing process as AgNPs not allows the tissue adherence and thereby helps in healing process [45, 46]. The antimicrobial dressing materials developed through the tethering of the AgNPs can be easily use for biomedical application.

Although, the AgNPs are broad spectrum antimicrobials, it is hard to conclude using clinical and laboratory control strains that it can be substitutes the antibiotics as their toxicity is still a big challenge for their clinical and biomedical applications. Furthermore, there are few controversies related to safe use of AgNPs in human diseases treatment and health care. In the present study, use of only three MDR strains may be inadequate and in future we will conduct the study with few more MDR strains. Nevertheless, the study provides the baseline information to develop the prototype for antimicrobial medical materials.

## Conclusion

Macerating enzymes are good reductant of AgNO_3_ for in vitro synthesis of silver nanoparticles. The adopted process was environment-friendly, simple, rapid, and easy to perform. Bioformulated AgNPs were well dispersed, small in size, elliptical and stable over a year suggest promising application in the biomedical and healthcare sector. Synthesized AgNPs showed antimicrobial activity against various non-MDR and MDR bacteria, yeast and molds. This study confirms the biologically synthesized AgNPs are safe and viable option against pathogens. So it is proved that the AgNPs has a great potential to be used as an antimicrobial agent. The nano-silver treated nylon thread can be useful especially for the stitching the surgery cuts to avoids the secondary infection.

## Additional file

**Additional file 1.** UV–Vis spectra of AgNPs synthesized at 37 °C, 60 °C, 80 °C and 90 °C temperature.

## Abbreviations

AgNO_3_: silver nitrate
AgNPs: silver nanoparticle
AMR: antimicrobials resistance
CLSI: Clinical and Laboratory Standards Institute
DLS: dynamic light scattering
EDX: energy-dispersive X-ray spectroscopy
ESBL: extended-spectrum beta-lactamases
fcc: face-centered cubic
JCPDS: Joint Committee on Powder Diffraction Standard
MDR: multi-drug resistant
MH: Mueller-Hinton agar
mm: millimeter
mM: millimolar
MRSA: methicillin-resistant *staphylococcus aureus*
NCCLS: National Committee for Clinical Laboratory Standards
NCIM: National Collection of Industrial Microorganisms
PDA: potato dextrose agar
PDR: pan drug resistance
SEM: scanning electron microscopy
TEM: transmission electron microscopy
WHO: World Health Organization
XDR: extensively drug-resistant
XRD: X-ray diffraction
ZOI: zone of inhibition
μg: microgram
μl: microliter
