# Supplementary material for: Enzyme-mediated formulation of stable elliptical silver nanoparticles tested against clinical pathogens and MDR bacteria and development of antimicrobial surgical thread
Source: Ann Clin Microbiol Antimicrob. 2017 May 16;16:39. doi: 10.1186/s12941-017-0216-y (PMC5434635; doi:10.1186/s12941-017-0216-y)
Supplement: Supplementary file 1 — Additional file 1. UV–Vis spectra of AgNPs synthesized at 37 °C, 60 °C, 80 °C and 90 °C temperature. [file 12941_2017_216_MOESM1_ESM.pdf]

Additional Figure

UV-Vis spectra of AgNPs synthesized at (A) 37 ºC (B) 60 ºC (C) 80 ºC and (D) 90 ºC temperature and AgNPs biosynthesis confirmed over a period of 2 to 8 hours. Tube image indicated the color changed in the reaction mixture after 8 hours treatment at each specified temperature.

A

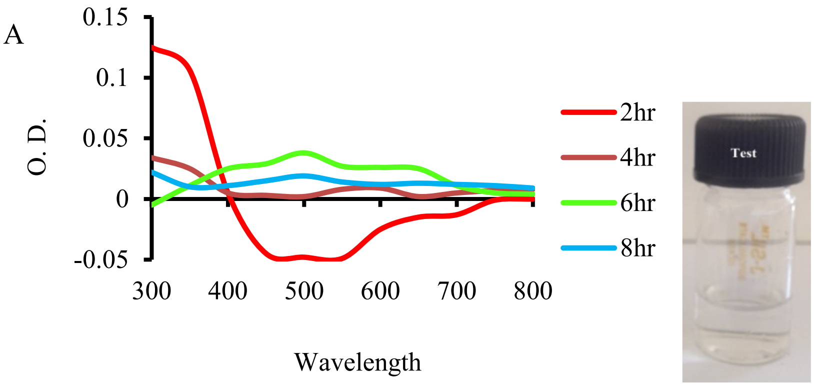

B

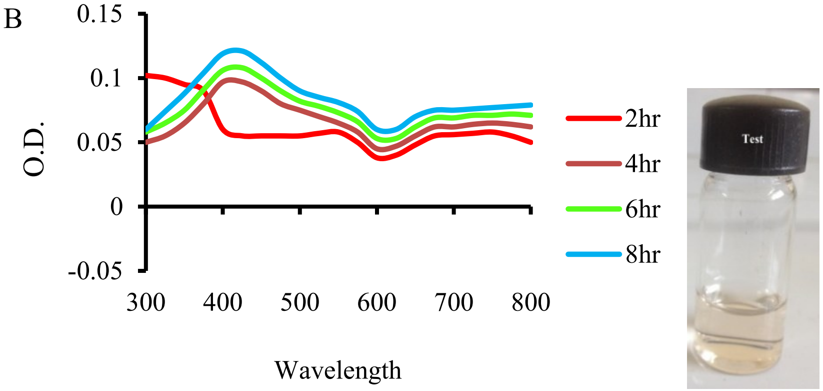

C

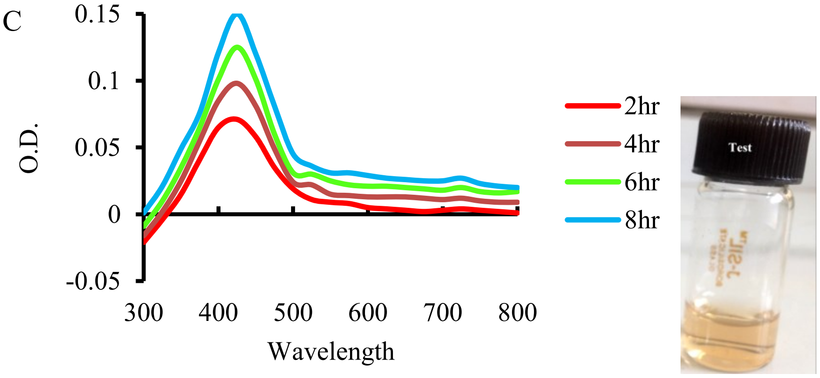

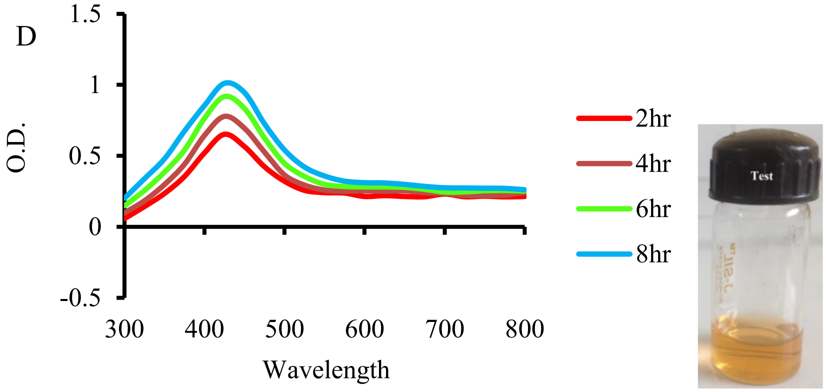
D
1.5
1
0.5
0
-0.5
O.D.
300
400
500
600
700
800
Wavelength
2hr
4hr
6hr
8hr
Test
